# Severe α_1_-antitrypsin deficiency associated with lower blood pressure and reduced risk of ischemic heart disease: a cohort study of 91,540 individuals and a meta-analysis

**DOI:** 10.1186/s12931-022-01973-3

**Published:** 2022-03-09

**Authors:** Sine Voss Winther, Dunia Ahmed, Suzan Al-Shuweli, Eskild Morten Landt, Børge Grønne Nordestgaard, Niels Seersholm, Morten Dahl

**Affiliations:** 1 Department of Clinical Biochemistry, Zealand University Hospital, Køge, Denmark; 2 Department of Clinical Biochemistry, Herlev-Gentofte University Hospital, Herlev, Denmark; 3 The Copenhagen General Population Study, Herlev-Gentofte University Hospital, Herlev, Denmark; 4 Department of Clinical Medicine, Copenhagen University, Copenhagen, Denmark; 5 Department of Pulmonary Medicine, Herlev-Gentofte University Hospital, Gentofte, Denmark

**Keywords:** α_1_-Antitrypsin deficiency, Blood pressure, Cardiovascular disease, Epidemiology, Genetics, Lipids, SERPINA1

## Abstract

**Background:**

Increased elastase activity in α_1_-antitrypsin deficiency may affect elasticity of the arterial walls, and thereby blood pressure and susceptibility to cardiovascular disease. We hypothesized that severe α_1_-antitrypsin deficiency is associated with reduced blood pressure and susceptibility to cardiovascular disease.

**Methods:**

We genotyped 91,353 adults randomly selected from the Danish general population and 187 patients from the Danish α_1_-Antitrypsin Deficiency Registry and recorded baseline blood pressure, baseline plasma lipids and cardiovascular events during follow-up. 185 participants carried the ZZ genotype, 207 carried the SZ genotype and 91,148 carried the MM genotype.

**Results:**

α_1_-Antitrypsin deficiency was associated with decreases in blood pressure of up to 5 mmHg for systolic blood pressure and up to 2 mmHg for diastolic blood pressure, in ZZ vs SZ vs MM individuals (trend test, *P*’s ≤ 0.01). Plasma triglycerides and remnant cholesterol were reduced in ZZ individuals compared with MM individuals (*t*-test, *P*’s < 0.001). α_1_-Antitrypsin deficiency was associated with lower risk of myocardial infarction (trend test *P* = 0.03), but not with ischemic heart disease, ischemic cerebrovascular disease or hypertension (trend test, *P*’s ≥ 0.59). However, when results for ischemic heart disease were summarized in meta-analysis with results from four previous studies, individuals with versus without α_1_-antitrypsin deficiency had an odds ratio for ischemic heart disease of 0.66 (95% CI:0.53–0.84).

**Conclusions:**

Individuals with severe α_1_-antitrypsin deficiency have lower systolic and diastolic blood pressure, lower plasma triglycerides and remnant cholesterol, reduced risk of myocardial infarction, and a 34% reduced risk of ischemic heart disease.

**Supplementary Information:**

The online version contains supplementary material available at 10.1186/s12931-022-01973-3.

## Background

α_1_-Antitrypsin is a serine protease inhibitor, which inhibits a series of proteases, including neutrophil elastase, thereby protecting elastic tissue in the lungs [1, 2]. The coding gene termed SERPINA1 is located on chromosome 14, and more than 150 variations of this gene have been identified [2, 3]. The most common combination of the gene variations is the MM genotype, also termed the wildtype genotype, which is associated with a normal plasma level and function of α_1_-antitrypsin. The S- and Z variants cause different degrees of α_1_-antitrypsin deficiency, the ZZ genotype being the most severe with a reduction in circulating α_1_-antitrypsin of up to 85% [1]. α_1_-Antitrypsin deficiency is mostly known to result in an early onset of chronic obstructive pulmonary disease (COPD), due to the lack of inhibition of neutrophil elastase, and liver cirrhosis due to polymerization of mutant α_1_-antitrypsin in the endoplasmic reticulum, resulting in hepatocyte damage [1–3].

Studies have suggested that the increased elastase activity over time also affects the elastic properties of the arterial walls [4, 5]. The larger blood vessels contain large amounts of elastin in tunica media, contributing to the distensibility, resilience and recoil of the vessel wall [6, 7]. Our hypothesis is that the increased activity of neutrophil elastase in α_1_-antitrypsin deficiency changes the elasticity of the vessel wall, resulting in decreased resistance and thereby a lower blood pressure [6]. Reduced blood pressure in individuals with α_1_-antitrypsin deficiency could contribute to a reduced risk of cardiovascular disease, for example ischemic heart disease and stroke [5].

We hypothesized that α_1_-antitrypsin deficiency is associated with reduced blood pressure and susceptibility to cardiovascular disease in individuals in the general population. To test this hypothesis, we genotyped 91,353 adults from the Copenhagen General Population Study and 187 patients from the Danish α_1_-Antitrypsin Deficiency Registry and recorded baseline blood pressure, baseline plasma lipids and cardiovascular disease during follow-up as outcomes. As a secondary aim, we also performed a meta-analysis of previous studies examining the risk of ischemic heart disease in individuals with α_1_-antitrypsin deficiency.

## Methods

### Participants

We included participants from The Copenhagen General Population Study and the Danish α_1_-Antitrypsin Deficiency Registry. Cases from the two studies did not differ significantly for any of the outcomes studied (*P*’s for interaction ≥ 0.1), and were therefore combined yielding a total population of 91,540, within which 185 were ZZ individuals, 207 were SZ individuals, and 91,148 were MM individuals. Individuals with MZ, SS, and MS α_1_-antitrypsin deficiencies were excluded from the analysis (n = 10,965). However, if these individuals were included in the analysis the results were similar to those presented.

The Copenhagen General Population Study was initiated in 2003 with ongoing enrollment [8]. Individuals were randomly selected on the basis of the national Danish Civil Registration System to reflect the adult white population aged 20 to 100 years. Data were obtained from a questionnaire and a physical health examination, and blood samples were drawn for biochemical analyses and DNA extraction. We included 91,353 consecutive participants from this study, within which 48 carried the ZZ genotype, 157 carried the SZ genotype, and 91,148 carried the MM genotype.

The Danish α_1_-Antitrypsin Deficiency Registry was initiated in 1978 and includes patients diagnosed with severe α_1_-antitrypsin deficiency. Patients from the registry were examined from August 31st 2009 to October 6th 2009 as part of the Copenhagen General Population Study undergoing the exact same investigations as all other participants in this survey. We included 187 participants from the registry, within which 137 carried the ZZ genotype and 50 carried the SZ genotype.

### Endpoints

Blood pressure was measured by trained technicians on the left upper arm after 5 min rest with the individual in the sitting position. An automatic Digital Blood Pressure Monitor (Kivex, Hørsholm, Denmark) with a cuff measuring 22 × 32 cm was used; if the circumference of the upper arm was > 46 cm, we used a cuff measuring 32 × 45 cm. All participants had their blood pressure measured within the 3-7 pm time interval. From March 2009 and onward, blood pressure was measured on both arms and legs with the participants resting in supine position. Systolic blood pressure of the posterior tibial artery or the dorsalis pedis artery was obtained by a handheld Doppler. Ankle-brachial index (ABI) was calculated as the lowest ankle systolic blood pressure level divided by the right arm brachial systolic blood pressure level. Plasma α_1_-antitrypsin, lipids, and liver markers were measured by standard hospital assays; remnant cholesterol was calculated as total cholesterol minus low-density lipoprotein (LDL) cholesterol minus high-density lipoprotein (HDL) cholesterol. Ischemic heart disease (ICD8: 410–414, ICD10: I20-I25), ischemic cerebrovascular disease (ICD8: 432–435, ICD10: I63-I64, G45), myocardial infarction (ICD8: 410, ICD10: I21-I22), and COPD (ICD8: 491-492, ICD10:J41-J44) were defined as hospital admissions with the diagnosis as the primary discharge diagnosis, obtained from the national Danish Patient Registry. Hypertension was systolic blood pressure ≥ 140 mmHg, diastolic blood pressure ≥ 90 mmHg, and/or current use of antihypertensive medication [9].

### Genotyping

We genotyped for the S (Glu264Val) and Z (Glu342Lys) variants by using TaqMan-based PCR assays and an ABI PRISM 7900HT Sequence Detection system (Applied Biosystems, Foster City, CA). The following primers and probes were used: Exon 3 (S allele), forward primer CTGATGAAATACCTGGGCAATGC, reverse primer TGGTGATGATATCGTGGGTGAGT, VIC probe CACCTGGAAAATGA, and 6-FAM probe CACCTGGTAAATGA; exon 5 (Z allele), forward primer CTTACAACGTGTCTCTGCTTCTCT, reverse primer

CAAAGGGTTTGTTGAACTTGACCTC, VIC probe CTTCAGTCCCTTTCTCGTCGA, and 6-FAM probe TTCAGTCCCTTTCTTGTCGA.

### Statistical analysis

All statistical analyses were performed using STATA version 12.1 (College Station, Texas, USA). Student’s t-test was used for continuous variables, and Pearson’s χ^2^ test for categorical data. Plasma triglycerides, HDL cholesterol, lipoprotein (a), remnant cholesterol, and bilirubin were logarithmically transformed due to positively skewed distributions. Cox-regression adjusting for age, sex, smoking status and COPD was used to assess risk of cardiovascular disease prospectively in individuals with versus without α_1_-antitrypsin deficiency, and logistic regression adjusting for age, sex, smoking status and COPD was used to asses risk of hypertension cross-sectionally in individuals with versus without α_1_-antitrypsin deficiency. Interactions between genotype and sex, age, smoking, and COPD on cardiovascular outcomes were tested in logistic regression models. Because outcomes did not differ significantly according to sex (*P’s* > 0.05), combined results were presented. In sensitivity analyses, multiple logistic regression was performed using stepwise forward inclusion, where continuous variables were dichotomized by the median value, and genotype was added to the final model irrespective of significance. The forward inclusion method adds variables one by one to the regression model, based on the overall significance of the test statistic, i.e. minimizing the p-value of the likelihood ratio chi-squared (LR χ^2^) test statistic. In sensitivity analyses, the associations between plasma α_1_-antitrypsin and systolic blood pressure, and cardiovascular outcomes were analyzed using binned scatter plots with R^2^ as a measure of the correlation.

### Meta-analysis

We searched PubMed up until October 1^st^ 2021 querying for (“alpha-1-antitrypsin-deficiency” OR “alpha 1 antitrypsin deficiency” OR AATD OR antitrypsin OR SERPINA1) AND (“ischemic heart disease” OR IHD OR CVD OR “cardiovascular disease” OR stroke OR “ischemic stroke” OR “myocardial infarction” OR MI OR hypertension”) using MESH terms and free text. Reference lists were searched for additional relevant publications. 5 studies (the present study and [5, 10–12]) examining α_1_-antitrypsin deficiency in relation to ischemic heart disease as the primary endpoint were included. Severe α_1_-antitrypsin deficiency was either physician diagnosed α_1_-antitrypsin deficiency with an ICD10 code (E88.0), self-reported physician diagnosed α_1_-antitrypsin deficiency, or a diagnosis of α_1_-antitrypsin deficiency based on PCR or isoeletric focusing. Because previous studies were based on SZ and ZZ combined we also pooled data on SZ and ZZ in our own dataset. Myocardial infarction was used as the primary endpoint in studies where both ischemic heart disease and myocardial infarction results were reported. Meta-analysis was conducted using the MIX 2.0 (Professional software for meta-analysis in Excel. Version 2.015. BiostatXL, 2016) using a random effects model. The meta-analysis was conducted according to the PRISMA guidelines [13].

### Results

Clinical characteristics of individuals with ZZ and SZ α_1_-antitrypsin deficiency versus MM individuals in the population are shown in Table 1. Individuals with ZZ α_1_-antitrypsin deficiency were younger, had reduced values of plasma α_1_-antitrypsin and FEV1/FVC, were less likely current smokers, and had more often COPD compared with MM individuals. Individuals with SZ α_1_-antitrypsin deficiency vs MM individuals had reduced plasma α_1_-antitrypsin and had more often COPD, but did not differ in any other clinical characteristics. Use of antihypertensive medication did not differ between individuals with ZZ or SZ α_1_-antitrypsin deficiency versus MM individuals.

**Table 1:** Characteristics of participants according to α_1_-antitrypsin genotype

Genotype	MM	SZ	ZZ
Women/men	50,206/40,942	124/83	95/90
Age, yrs	57.8 ± 13.1	56.7 ± 13.3	54.8 ± 13.1^†^
Body mass index, kg/m^2^	26.2 ± 4.3	26.3 ± 4.7	26.2 ± 5.2
Plasma α_1_-antitrypsin, µmol/L	24.0 ± 4.1	10.4 ± 2.0^‡^	4.7 ± 4.8^‡^
FEV1/FVC, %	77.4 ± 15.9	76.5 ± 10.6	60.3 ± 19.3^‡^
Smoking, no. (%)			
Never smoker	35,533 (40.6)	84 (42.9)	73 (41.4)
Ex smoker	36,119 (41.3)	82 (41.6)	89 (51.7)
Current smoker	15,861 (18.1)	31 (15.8)	12 (6.9)^†^
Chronic Obstructive Pulmonary Disease, no. (%)	5,137 (5.7)	25 (12.2)^‡^	100 (54.1)^‡^
Antihypertensive medication, no. (%)	17,898 (19.7)	45 (22.0)	41 (22.7)

Values presented as mean ± SD or number (%). ^†^*P* value < 0.01, ^‡^*P*value < 0.001 on Student’s *t*-test or Pearson’s *χ*^2^ test versus MM genotype. *FEV1* forced expiratory volume in 1 s. *FVC* forced vital capacity

### Blood pressure

**Fig. 1:**
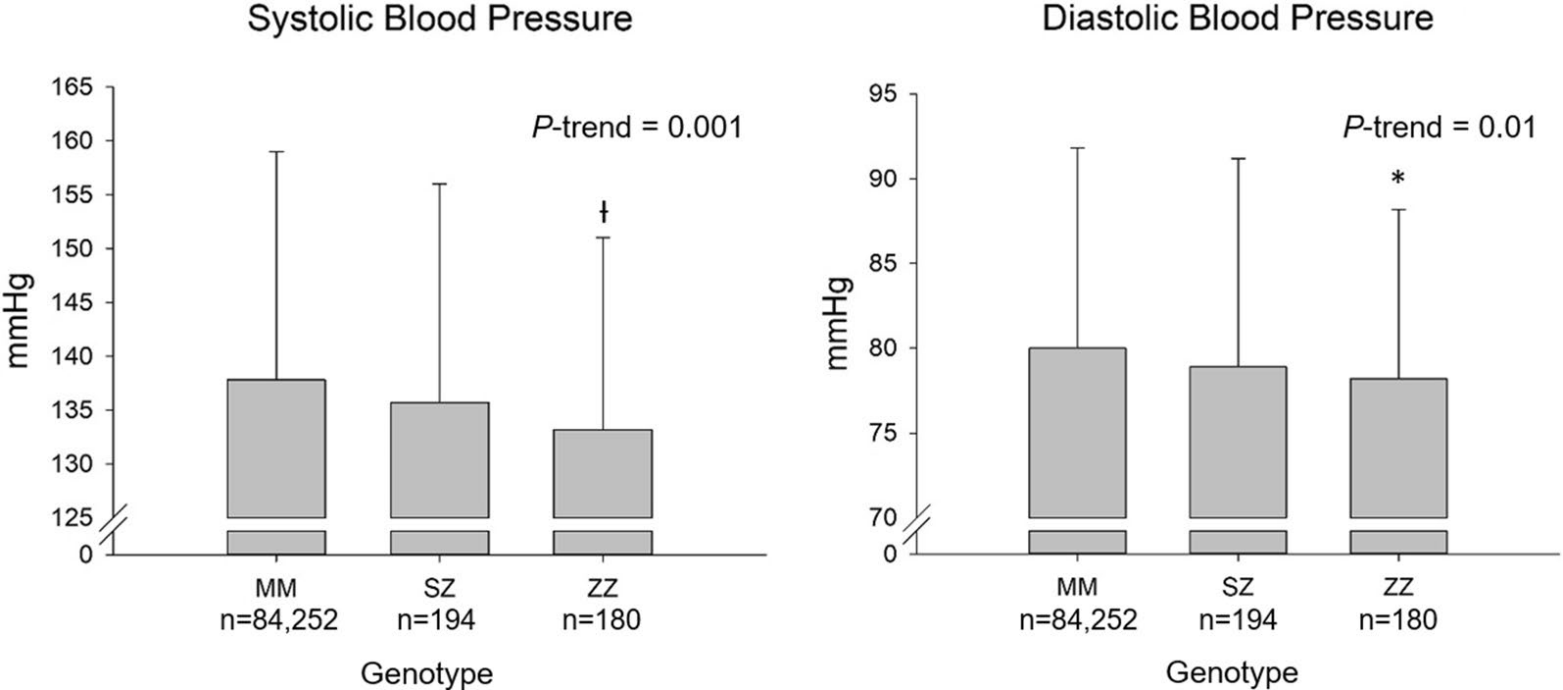
Blood pressure according to α_1_-antitrypsin deficiency genotype. Values are mean values with error bars representing SD´s. *P value < 0.05, ^†^*P* value < 0.01 versus MM genotype on Student’s *t*-test

Systolic blood pressure was lower as a function of α_1_-antitrypsin genotype (trend test MM-SZ-ZZ, *P* = 0.001, Fig. 1): individuals with ZZ and SZ α_1_-antitrypsin deficiencies had systolic blood pressure of 133 mmHg (t-test vs MM, *P* = 0.003) and 136 mmHg (*P* = 0.17), respectively, compared with 138 mmHg in MM individuals. Diastolic blood pressure was also reduced as a function of α_1_-antitrypsin genotype (trend test MM-SZ-ZZ, *P* = 0.01, Fig. 1): individuals with ZZ and SZ α_1_-antitrypsin deficiencies had diastolic blood pressure of 78.2 mmHg (t-test vs MM, *P* = 0.04) and 78.9 mmHg (*P* = 0.20), respectively, compared with 80.0 mmHg in MM individuals (Fig. 1). If these results were adjusted for age, sex, and smoking status similar results to those presented were seen (trend test MM-SZ-ZZ, *P*’s ≤ 0.04). When measuring blood pressure and ankle-brachial index in the supine position in a subgroup of individuals, similar results were seen for systolic blood pressure (trend test MM-SZ-ZZ, *P* = 0.003), but not for diastolic blood pressure or ankle-brachial index (*P*’s ≥ 0.44, Additional file 1: Figs. S1 and S2): individuals with ZZ and SZ α_1_-antitrypsin deficiencies had supine systolic blood pressure of 137 mmHg (t-test vs MM, *P* = 0.006) and 138 mmHg (*P* = 0.006), respectively, compared with 142 mmHg in MM individuals.

### Lipids and liver parameters

Individuals with ZZ α_1_-antitrypsin deficiency vs MM individuals had 0.3 mmol/L lower plasma triglycerides (t-test: *P* < 0.001) and 0.1 mmol/L lower remnant cholesterol (*P* < 0.001), and higher values of plasma ALAT (20% higher; *P* < 0.001), bilirubin (10% higher; *P* < 0.01) and alkaline phosphatase (5% higher; *P* < 0.05) (Table 2). Individuals with SZ α_1_-antitrypsin deficiency compared with MM individuals also had higher plasma ALAT (*P* < 0.01) and alkaline phosphatase (*P* < 0.001), but did not differ in any other plasma lipid or liver parameters. If these results were adjusted for age, sex, and smoking status similar results to those presented were seen (trend test, *P*’s < 0.007).

**Table 2:** Plasma lipids and liver parameters according to α_1_-antitrypsin genotype

Genotype	MM (n = 91,148)	SZ (n = 207)	ZZ (n = 185)
Plasma lipids			
Total cholesterol, mmol/L	5.6 ± 1.1	5.6 ± 1.0	5.5 ± 1.0
HDL cholesterol, mmol/L	1.6 (1.2–1.9)	1.5 (1.2–1.9)	1.6 (1.3–2.0)
LDL cholesterol, mmol/L	3.2 ± 1.0	3.3 ± 0.9	3.2 ± 0.8
Remnant cholesterol, mmol/L	0.6 (0.4–0.9)	0.7 (0.5–0.9)	0.5 (0.4–0.7)^‡^
Triglycerides, mmol/L	1.4 (1.0–2.1)	1.4 (1.0–2.1)	1.1 (0.8–1.6)^‡^
Lipoprotein(a), mg/dL	11.7 (5.9–31.0)	12.1 (6.3–25.8)	13.5 (6.0–30.4)
Plasma liver parameters			
ALAT, U/L	23.6 ± 16.6	27.0 ± 14.8^†^	28.3 ± 16.4^‡^
Bilirubin, µmol/L	10 (8–13)	11 (8–13)	11 (9–14)^†^
Alkaline phosphatases, U/L	82.2 ± 25.8	89.7 ± 26.9^‡^	86.6 ± 25.9*
Lactate dehydrogenase U/L	158 ± 33.8	158 ± 39.4	163 ± 36.0

Values presented as mean ± SD or median (25 and 75 percentiles). *P value< 0.05, ^†^*P* value< 0.01, and ^‡^*P* value< 0.001 versus MM on Student’s *t*-test. *ALAT* alanine aminotransferase

### Cardiovascular disease

**Fig. 2:**
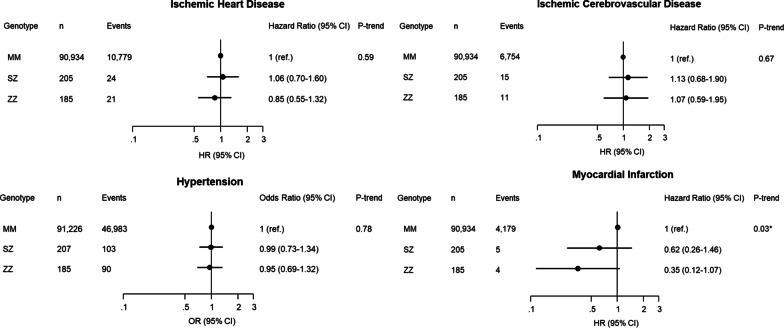
Risk of cardiovascular disease according to α_1_-antitrypsin deficiency genotype. Regression models were adjusted for age, sex,smoking status and COPD. Ischemic heart disease = ICD8: 410–414; ICD10: I20-I25. Ischemic cerebrovascular disease = ICD8: 423–435; ICD10: I63, I64, G45. Hypertension = systolic blood pressure ≥ 140, diastolic blood pressure ≥ 90 and/or use of current antihypertensive medication. Myocardial infarction = ICD8: 410; ICD10: I21-I22

α_1_-Antitrypsin deficiency was associated with lower risk of myocardial infarction (trend test, MM-SZ-ZZ, *P* = 0.03), but there were no significant associations of α_1_-antitrypsin deficiency with risks of ischemic heart disease, ischemic cerebrovascular disease or hypertension (trend test, *P*’s ≥ 0.59) (Fig. 2). In sensitivity analysis, identifying risk factors for cardiovascular outcomes from a stepwise forward approach, α_1_-antitrypsin deficiency remained associated with myocardial infarction (odds ratio, 0.38 and 95%CI: 0.18–0.77), while there were no associations with ischemic heart disease, ischemic cerebrovascular disease, or hypertension (Additional file 1: Fig. S3). There were no significant interactions between α_1_-antitrypsin deficiency and sex, age, smoking or COPD on cardiovascular outcomes (Additional file 1: Fig. S4). Plasma α_1_-antitrypsin was positively correlated with all cardiovascular outcomes studied (Additional file 1: Fig. S5).

**Fig. 3:**
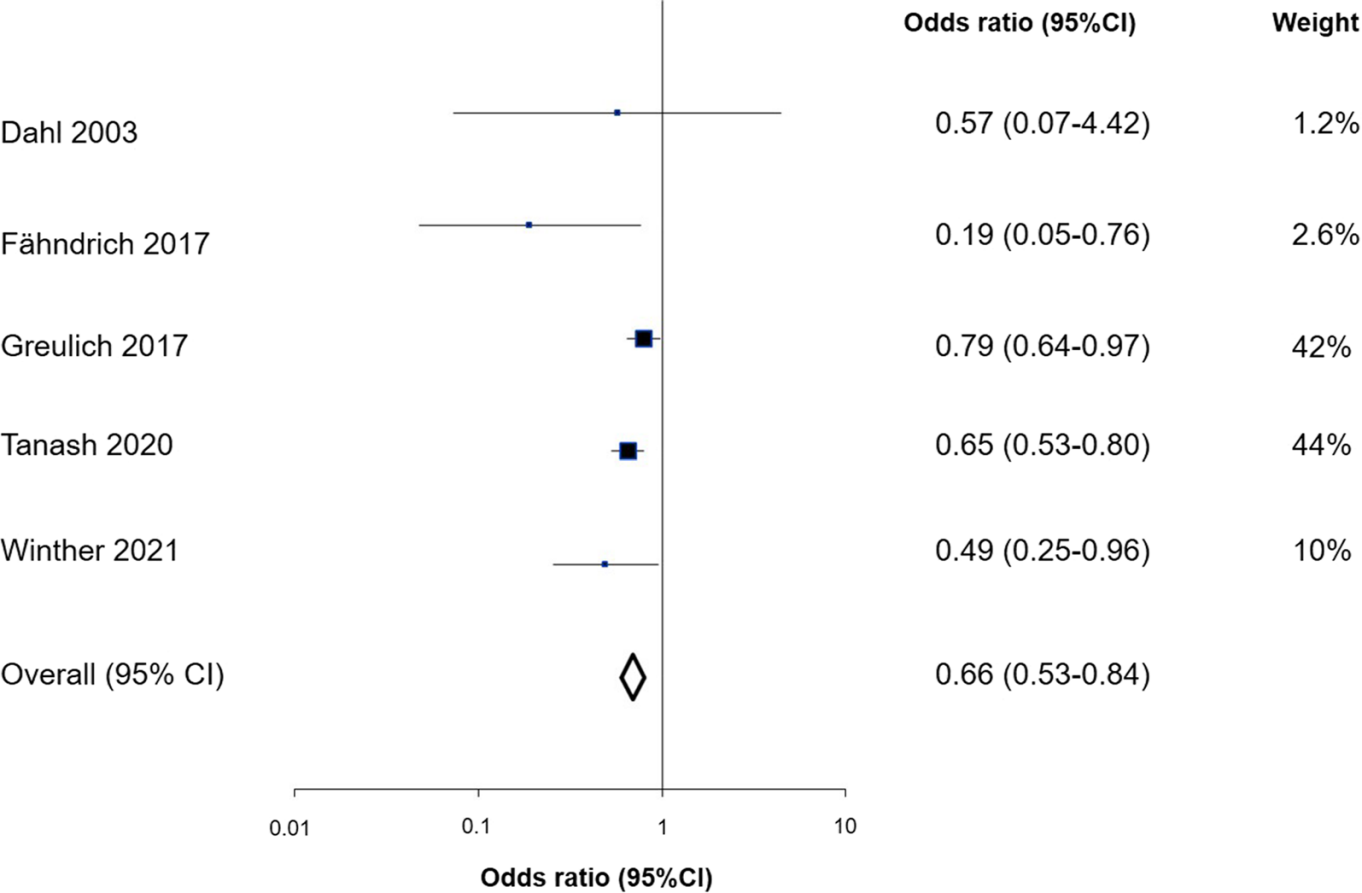
Case–control and cross-sectional studies of ischemic heart disease in individuals with α_1_-antitrypsin deficiency. Box sizes are proportional to inverse-variance weights. Lines represent 95% confidence intervals

When the results for ischemic heart disease were summarized in meta-analysis with results from four previous studies, individuals with versus without α_1_-antitrypsin deficiency had an odds ratio for ischemic heart disease of 0.66 (95% CI, 0.53 to 0.84) (Fig. 3). This meta-analysis was not characterized by heterogeneity between the included studies (*P* = 0.18) and provided robust summary odds ratios in sensitivity analyses (Additional file 1: Fig. S6). When each study was removed one at a time and the summary odds ratio recalculated, all the recalculated summary odds ratios lay within the 95% CI of the main results.

## Discussion

In this large Danish cohort study, we evaluated blood pressure, plasma lipids, and risk of cardiovascular disease in individuals with α_1_-antitrypsin deficiency vs MM individuals. The results revealed that individuals with severe α_1_-antitrypsin deficiency had 2 and 5 mmHg lower diastolic and systolic blood pressure, 0.3 mmol/L lower plasma triglycerides, 0.1 mmol/L lower remnant cholesterol, reduced risk of myocardial infarction, and a 34% reduced risk of ischemic heart disease in meta-analysis.

In the current analysis and in the subgroup analysis, we confirm our previous findings in the Copenhagen City Heart Study of reduced systolic blood pressure in individuals with α_1_-antitrypsin deficiency [5]. Compared to this previous study [5], reduced systolic blood pressure was replicated in two different settings in the current study with blood pressure measured respectively in the sitting and supine positions. Other previous but smaller studies found no or a similar association between reduced blood pressure and α_1_-antitrypsin deficiency [4, 14, 15], while Senn et al. identified a positive association between α_1_-antitrypsin level and systolic blood pressure [16], as was also observed for systolic blood pressure and for cardiovascular outcomes in the present study (Figure S5). Studies of aortic stiffness in individuals with α_1_-antitrypsin deficiency or humans receiving pancreatic elastase therapy have also been performed but with differing results [17–20]. If our results show the true magnitude of blood pressure reduction in α_1_-antitrypsin deficiency, the reduction of 5 mmHg in systolic blood pressure may not be trivial as it is comparable to the minimal clinically important difference of 2 mmHg proposed for systolic blood pressure in previous studies [21].

In line with reductions of 5 and 2 mmHg in systolic and diastolic blood pressures in the present study, individuals with α_1_-antitrypsin deficiency also had reduced risk of myocardial infarction and a 0.66 fold reduced risk of ischemic heart disease in meta-analysis. Greulich et al. showed that patients with α_1_-antitrypsin deficiency had a lower prevalence of ischemic heart disease when compared to age and gender matched controls with known chronic obstructive pulmonary disease [11], and Fähndrich et al. found an association between α_1_-antitrypsin deficiency and a reduced frequency of ischemic heart disease when looking at patients with α_1_-antitrypsin deficiency with and without augmentation therapy [10]. Two other previous studies also found associations of reduced risk of ischemic heart disease with α_1_-antitrypsin deficiency, but were not included in the meta-analysis due to lack of data [22, 23]. The finding of reduced ischemic heart disease risk in individuals with α_1_-antitrypsin deficiency in the meta-analysis is also in line with research by Tanash et al. showing a reduced mortality from ischemic heart disease as well as no other increased mortality with regard to stroke, aorta dissection, or cardiovascular disease in individuals with α_1_-antitrypsin deficiency [24].

Individuals with ZZ α_1_-antitrypsin deficiency in the current study had lower levels of plasma triglycerides and remnant cholesterol compared to MM controls, confirming previous results by Hamesch et al. [25], and Fähndrich et al. [10]. The underlying mechanism for this could be an increased impairment of normal liver functions in ZZ individuals as suggested by Hamesch et al., which accords well with the simultaneously higher liver blood parameters in these individuals in [25] and the present study. The finding of lower triglyceride and remnant cholesterol levels in individuals with ZZ α_1_-antitrypsin deficiency could perhaps also contribute to the reduced risk of ischemic heart disease observed in individuals with α_1_-antitrypsin deficiency in the present study.

Strengths of our study include a randomly chosen sample of an ethnically homogenous population, and blinding of investigators to risk factor status and the clinical outcomes investigated. A potential limitation of our study is that some of the individuals suffering from α_1_-antitrypsin deficiency and severe disease may not have attended the physical examination and participated in the study. Hardy–Weinberg equilibrium calculation suggested that 31 ZZ individuals were not included in the sample from the Copenhagen General Population Study; however, we do not think that this substantially influenced the results as sensitivity analysis showed almost similar results in the two study samples separately (*P*’s for interaction ≥ 0.1, Additional file 1: Fig. S7 for systolic blood pressure). Although the finding of reduced systolic blood pressure in individuals with α_1_-antitrypsin deficiency was replicated in ethnically homogenous and independent studies of the Danish population ([5], the present study), it would of course be desirable if the finding is replicated in additional large population studies in the future. Fähndrich et al. [10] questioned whether their findings of a lower prevalence of cardiovascular diagnoses in patients with α_1_-antitrypsin deficiency was due to the use of augmentation therapy or the α_1_-antitrypsin deficiency genotype itself. Augmentation therapy was not available in Denmark at the time of data collection. Our investigation thus supports that the α_1_-antitrypsin deficiency genotype is protective against ischemic heart disease, perhaps through the reduction in risk factors like systolic and diastolic blood pressure, plasma triglycerides and remnant cholesterol, rather than through augmentation therapy. Further studies are needed with regards to the possible molecular mechanisms underlying the observed reductions in blood pressure, plasma lipids, and ischemic heart disease risk in individuals with α_1_-antitrypsin deficiency.

## Conclusions

In conclusion, individuals with α_1_-antitrypsin deficiency had reductions of 2 and 5 mmHg in diastolic and systolic blood pressure, reduced risk of myocardial infarction, and a 34% lower risk of ischemic heart disease. The study also revealed reductions of plasma triglycerides and remnant cholesterol levels in ZZ vs MM individuals, and a dose–response relation between severe α_1_-antitrypsin deficiency and reduced blood pressure in the population.

## Supplementary Information

**Additional file 1.** **Figure S1.** Supine blood pressure according to α_1_-antitrypsin deficiency genotype. **Figure S2.** Ankle-brachial index according to α_1_-antitrypsin deficiency genotype. **Figure S3.** Risk of cardiovascular disease according to α_1_-antitrypsin deficiency genotype by step-up logistic regression. **Figure S4.** Risk of cardiovascular disease according to α_1_-antitrypsin deficiency genotype, stratified by sex, age, smoking status and COPD. **Figure S5.** Binscatter plots of systolic blood pressure, ischemic heart disease, ischemic cerebrovascular disease, or hypertension versus plasma α_1_-antitrypsin partitioned into 20 bins. **Figure S6.** Exclusion sensitivity plot. **Figure S7.** Systolic blood pressure according to α_1_-antitrypsin deficiency genotype, stratified by study population.

## Data Availability

The datasets used and/or analysed during the current study are available from the corresponding author on reasonable request.

## Abbreviations

ALAT: Alanine aminotransferase
COPD: Chronic obstructive pulmonary disease
FEV1: Forced expiratory volume in 1 s
FVC: Forced vital capacity
HDL: High density lipoprotein
LDL: Low density lipoprotein
PCR: Polymerase chain reaction
