# Supplementary material for: Severe α1-antitrypsin deficiency associated with lower blood pressure and reduced risk of ischemic heart disease: a cohort study of 91,540 individuals and a meta-analysis
Source: Respir Res. 2022 Mar 9;23:55. doi: 10.1186/s12931-022-01973-3 (PMC8905778; doi:10.1186/s12931-022-01973-3)
Supplement: Supplementary file 1 — Additional file 1. Figure S1. Supine blood pressure according to α1-antitrypsin deficiency genotype. Figure S2. Ankle-brachial index according to α1-antitrypsin deficiency genotype. Figure S3. Risk of cardiovascular disease according to α1-antitrypsin deficiency genotype by step-up logistic regression. Figure S4. Risk of cardiovascular disease according to α1-antitrypsin deficiency genotype, stratified by sex, age, smoking status and COPD. Figure S5. Binscatter plots of systolic blood pressure, ischemic heart disease, ischemic cerebrovascular disease, or hypertension versus plasma α1-antitrypsin partitioned into 20 bins. Figure S6. Exclusion sensitivity plot. Figure S7. Systolic blood pressure according to α1-antitrypsin deficiency genotype, stratified by study population. [file 12931_2022_1973_MOESM1_ESM.doc]

**Additional file 1**

**Severe α_1_-Antitrypsin deficiency associated with lower blood pressure and reduced risk of ischemic heart disease: A cohort study of 91,540 Danish individuals and a meta-analysis**

Sine Voss Winther^1,4^, Dunia Ahmed^1^, Suzan Al-Shuweli^1^, Eskild Morten Landt^1^, Børge Grønne Nordestgaard^2-4^, Niels Seersholm^5^, Morten Dahl^1,3,4^

^1^Department of Clinical Biochemistry, Zealand University Hospital, Region Zealand, Denmark.

^2^Department of Clinical Biochemistry, Herlev-Gentofte University Hospital, Herlev, Denmark.

^3^The Copenhagen General Population Study, Herlev-Gentofte University Hospital, Herlev, Denmark.

^4^Department of Clinical Medicine, Copenhagen University, Copenhagen, Denmark.

^5^Department of Pulmonary Medicine, Herlev-Gentofte University Hospital, Gentofte, Denmark.


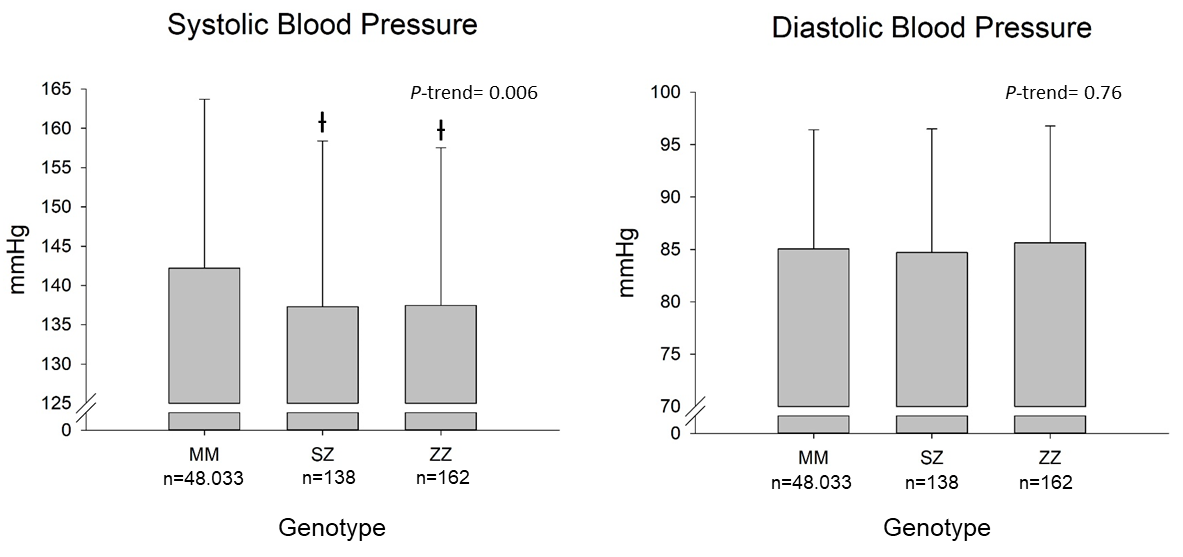


**Figure S1:** Supine blood pressure according to α_1_-antitrypsin deficiency genotype. Values are mean values with error bars representing SD’s. ^†^P<0.01 vs MM genotype on Student’s t-test.

Ankle-Brachial Index

*P*-trend= 0.44

**
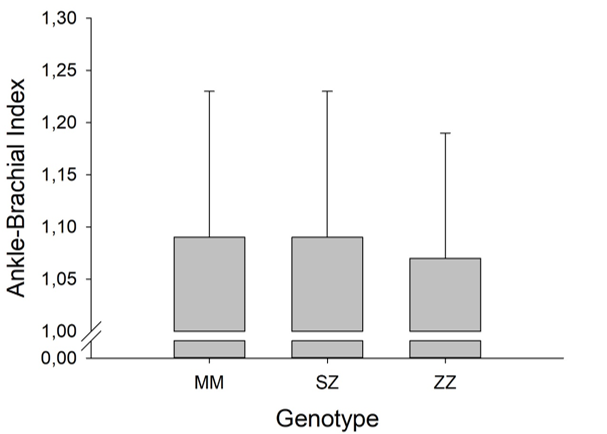
**

**Figure S2:** Ankle-brachial index according to α_1_-antitrypsin deficiency genotype. Values are mean values with error bars representing SD’s.

Ischemic Cerebrovascular Disease

Ischemic Heart Disease

| **Variable** | **OR** | **LCL** | **UCL** | **P-value** |
| --- | --- | --- | --- | --- |
| Genotype | 0.77 | 0.56 | 1.08 | 0.13 |
| Age | 3.38 | 3.22 | 3.54 | <0.001 |
| Type II Diabetes | 2.40 | 2.24 | 2.57 | <0.001 |
| Sex | 1.68 | 1.61 | 1.75 | <0.001 |
| COPD | 2.47 | 2.31 | 2.64 | <0.001 |
| BMI | 1.51 | 1.44 | 1.57 | <0.001 |
| LDL cholesterol | 0.73 | 0.70 | 0.76 | <0.001 |
|  |  |  |  |  |
| **LR chi2(7)** | 6650.2 |  |  |  |

| **Variable** | **OR** | **LCL** | **UCL** | **P-value** |
| --- | --- | --- | --- | --- |
| Genotype | 0.82 | 0.54 | 1.25 | 0.34 |
| Age | 4.23 | 3.97 | 4.52 | <0.001 |
| Type II Diabetes | 1.69 | 1.55 | 1.85 | <0.001 |
| Sex | 1.36 | 1.30 | 1.44 | <0.001 |
| COPD | 1.58 | 1.45 | 1.71 | <0.001 |
| SBP | 1.26 | 1.20 | 1.33 | <0.001 |
| LDL cholesterol | 0.80 | 0.76 | 0.84 | <0.001 |
| BMI | 1.09 | 1.03 | 1.15 | 0.002 |
|  |  |  |  |  |
| **LR chi2(8)** | 3727.9 |  |  |  |

Myocardial Infarction

Hypertension

| **Variable** | **OR** | **LCL** | **UCL** | **P-value** |
| --- | --- | --- | --- | --- |
| Genotype | 0.88 | 0.71 | 1.09 | 0.25 |
| Age | 3.77 | 3.67 | 3.88 | <0.001 |
| BMI | 2.06 | 2.00 | 2.12 | <0.001 |
| Type II Diabetes | 2.73 | 2.53 | 2.96 | <0.001 |
| Sex | 1.37 | 1.33 | 1.41 | <0.001 |
| COPD | 1.31 | 1.23 | 1.40 | <0.001 |
|  |  |  |  |  |
| **LR chi2(6)** | 15067.50 |  |  |  |

| **Variable** | **OR** | **LCL** | **UCL** | **P-value** |
| --- | --- | --- | --- | --- |
| Genotype | 0.38 | 0.18 | 0.77 | 0.007 |
| Age | 3.32 | 3.07 | 3.60 | <0.001 |
| Sex | 2.11 | 1.97 | 2.26 | <0.001 |
| Type II Diabetes | 2.22 | 2.02 | 2.44 | <0.001 |
| COPD | 2.07 | 1.88 | 2.27 | <0.001 |
| BMI | 1.55 | 1.45 | 1.67 | <0.001 |
| LDL cholesterol | 0.65 | 0.61 | 0.70 | <0.001 |
| Smoking Status | 1.53 | 1.41 | 1.64 | <0.001 |
|  |  |  |  |  |
| **LR chi2(8)** | 3412.7 |  |  |  |

**Figure S3:** Risk of cardiovascular disease according to α_1_-antitrypsin deficiency genotype by step-up logistic regression. Ischemic heart disease= ICD8: 410-414; ICD10: I20-I25. Ischemic cerebrovascular disease= ICD8: 423-435; ICD10: I63, I64, G45. Hypertension= systolic blood pressure≥140, diastolic blood pressure≥90 and/or use of current antihypertensive medication. Myocardial infarction= ICD8: 410; ICD10: I21-I22. OR= Odds ratio. LCL= Lower confidence interval limit. UCL= Upper confidence interval limit

**
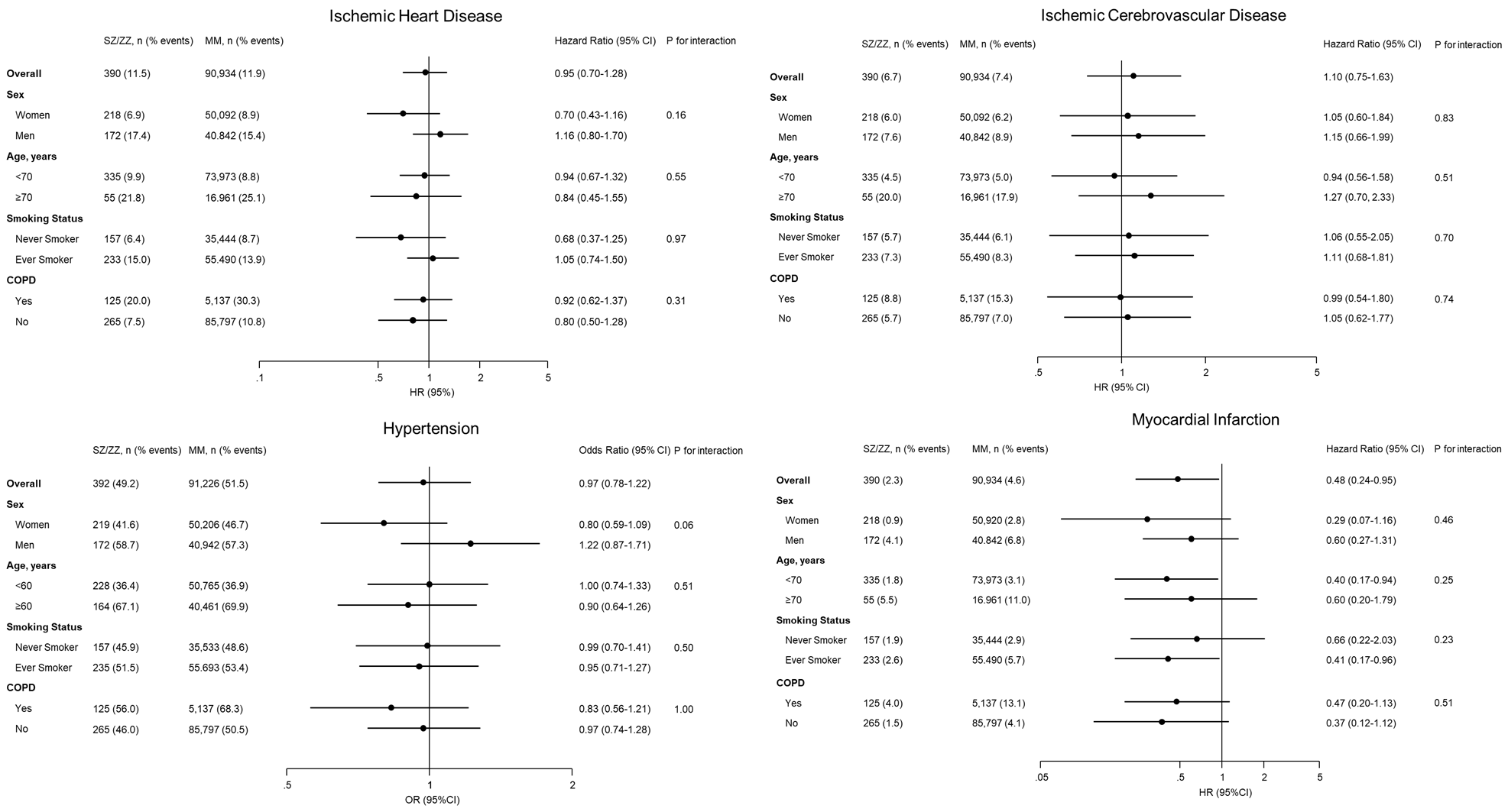
**

**Figure S4:** Risk of cardiovascular disease according to α_1_-antitrypsin deficiency genotype, stratified by sex, age, smoking status and COPD. Ischemic heart disease= ICD8: 410-414; ICD10: I20-I25. Ischemic cerebrovascular disease= ICD8: 423-435; ICD10: I63, I64, G45. Hypertension= systolic blood pressure≥140, diastolic blood pressure≥90 and/or use of current antihypertensive medication. Myocardial infarction= ICD8: 410; ICD10: I21-I22. CI= Confidence interval.

**
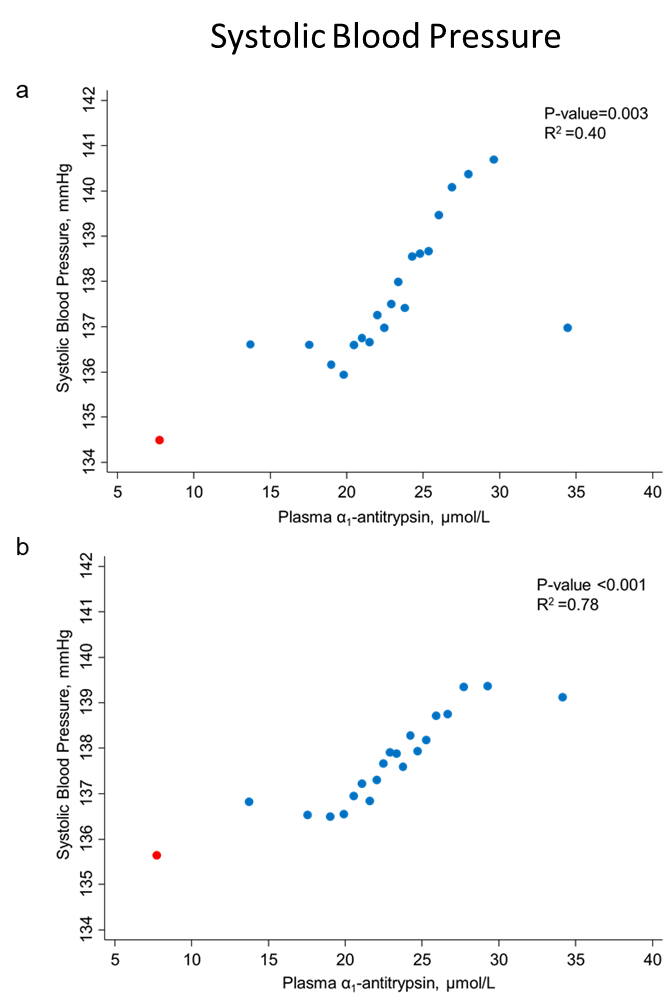
**

**Figure S5:** Binscatter plots of systolic blood pressure, ischemic heart disease, ischemic cerebrovascular disease, or hypertension versus plasma α_1_-antitrypsin partitioned into 20 bins. a) Unadjusted outcome/dependent attribute, b) outcome/dependent attribute adjusted using residuals from multiple linear regression models of the outcome vs. age, sex, smoking status and future COPD. Red dots correspond to the mean (a) or adjusted outcome mean (b) for the 392 individuals with SZ/ZZ genotype.


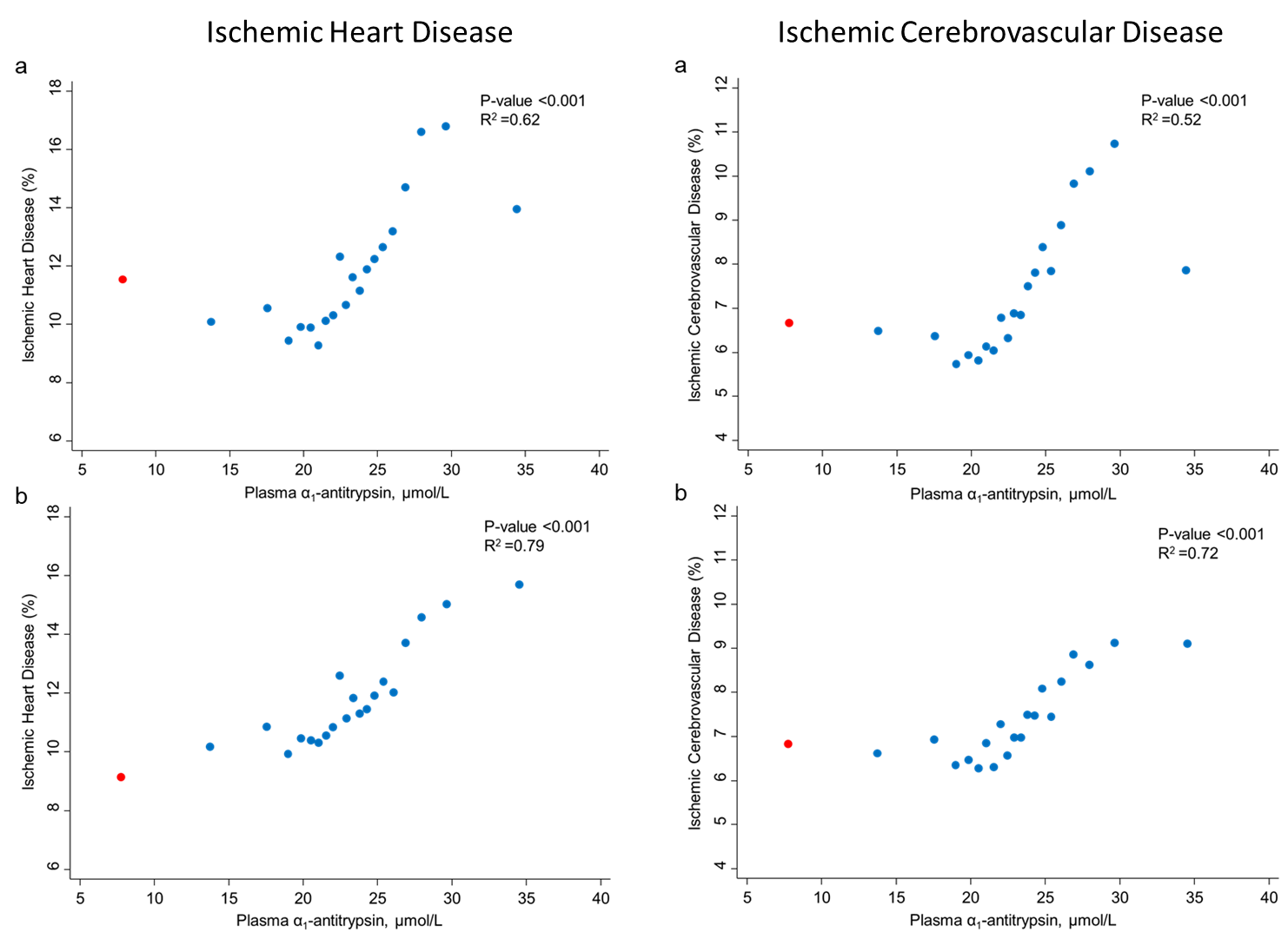


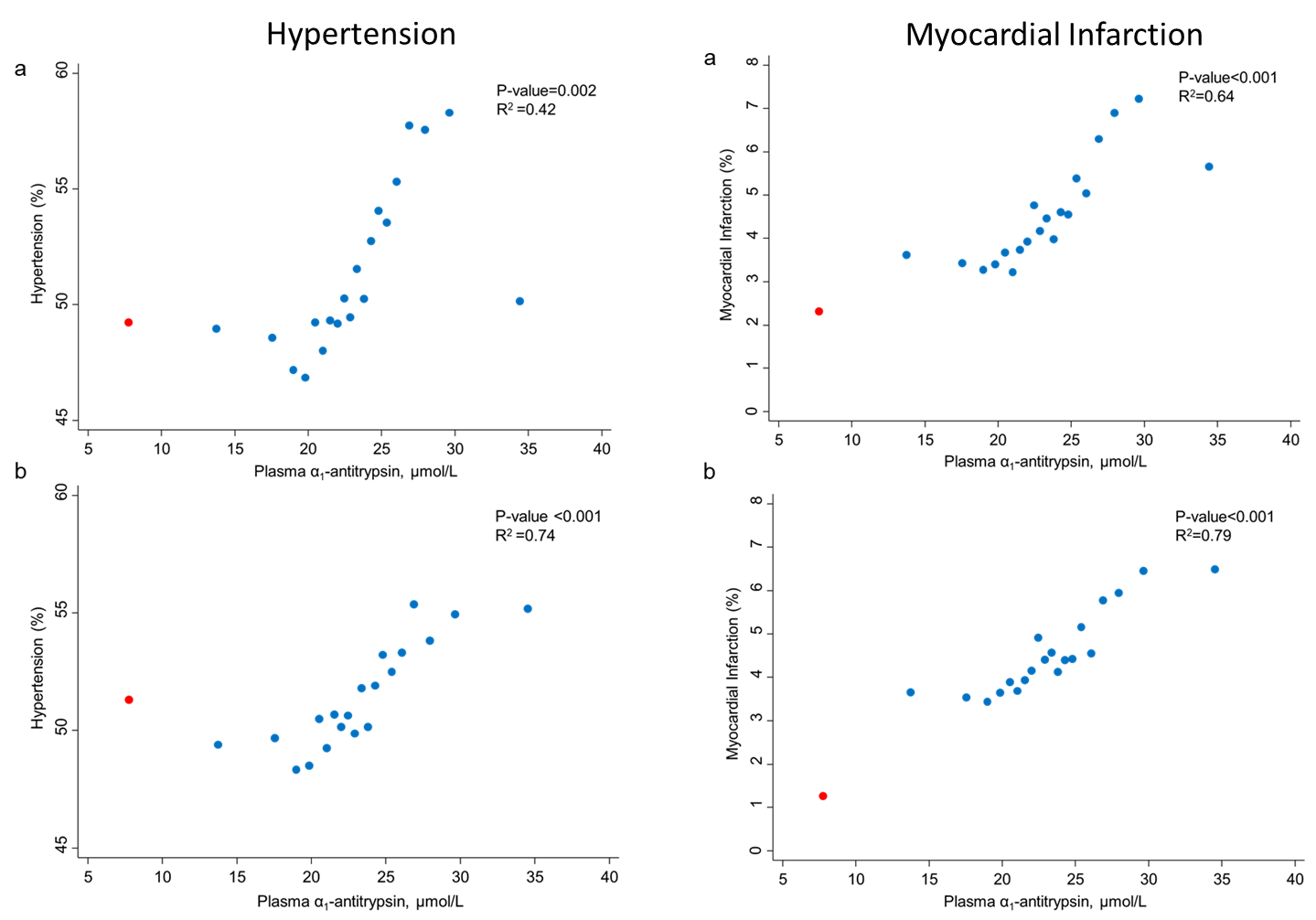


**Figure S6:** Exclusion sensitivity plot. Removal of each individual study did not significantly alter the summary odds ratio (fixed-effects meta-analysis).

**
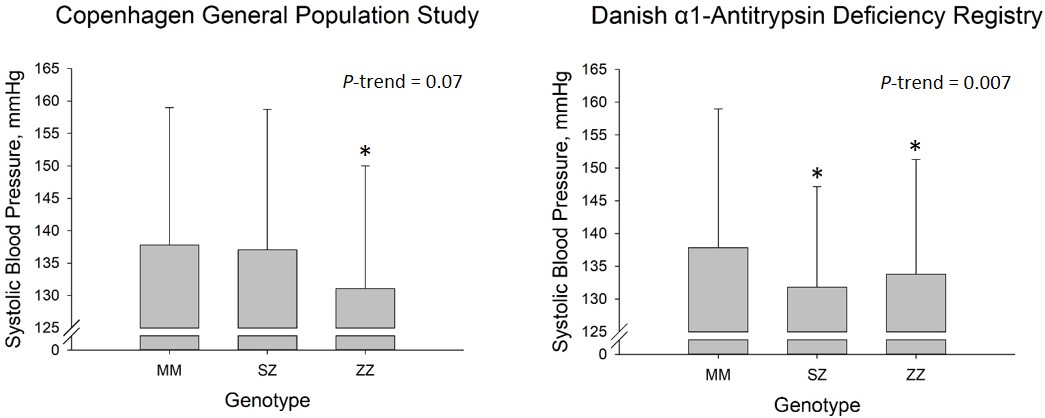
**

**Figure S7:** Systolic blood pressure according to α_1_-antitrypsin deficiency genotype, stratified by study population. Values are mean values with error bars representing SD’s. **P*<0.05 vs MM genotype on Student’s t-test.

STROBE Statement—Checklist of items that should be included in reports of ***cohort studies***

|  | Item No | Recommendation | Page no. |
| --- | --- | --- | --- |
| **Title and abstract** | 1 | (*a*) Indicate the study’s design with a commonly used term in the title or the abstract | Title |
|  |  | (*b*) Provide in the abstract an informative and balanced summary of what was done and what was found | Abstract, paragraph 2-4 |
| Introduction | | |  |
| Background/rationale | 2 | Explain the scientific background and rationale for the investigation being reported | Introduction, paragraph 1-2 |
| Objectives | 3 | State specific objectives, including any prespecified hypotheses | Introduction, paragraph 3 |
| Methods | | |  |
| Study design | 4 | Present key elements of study design early in the paper | Title and abstract |
| Setting | 5 | Describe the setting, locations, and relevant dates, including periods of recruitment, exposure, follow-up, and data collection | Methods, paragraph 2-4, 8 |
| Participants | 6 | (*a*) Give the eligibility criteria, and the sources and methods of selection of participants | Methods, paragraph 2-4 |
| Variables | 7 | Clearly define all outcomes, exposures, predictors, potential confounders, and effect modifiers. Give diagnostic criteria, if applicable | Methods, paragraph 5 |
| Data sources/ measurement | 8* | For each variable of interest, give sources of data and details of methods of assessment (measurement). Describe comparability of assessment methods if there is more than one group | Methods, paragraph 5-6 |
| Bias | 9 | Describe any efforts to address potential sources of bias | Discussion, paragraph 5 |
| Study size | 10 | Explain how the study size was arrived at | Methods, paragraph 2-4 |
| Quantitative variables | 11 | Explain how quantitative variables were handled in the analyses. If applicable, describe which groupings were chosen and why | Methods, paragraph 7 |
| Statistical methods | 12 | (*a*) Describe all statistical methods, including those used to control for confounding | Methods, paragraph 7 |
|  |  | (*b*) Describe any methods used to examine subgroups and interactions | Discussion, paragraph 5 |
|  |  | (*c*) Explain how missing data were addressed | N/A |
|  |  | (*d*) If applicable, describe analytical methods taking account of sampling strategy | N/A |
|  |  | (*e*) Describe any sensitivity analyses | Discussion, paragraph 5 |
| Results | | |  |
| Participants | 13* | (a) Report numbers of individuals at each stage of study—eg numbers potentially eligible, examined for eligibility, confirmed eligible, included in the study, completing follow-up, and analysed | Methods, paragraph 2-4 |
|  |  | (b) Give reasons for non-participation at each stage | N/A |
|  |  | (c) Consider use of a flow diagram | N/A |
| Descriptive data | 14* | (a) Give characteristics of study participants (eg demographic, clinical, social) and information on exposures and potential confounders | Results, paragraph 1 |
|  |  | (b) Indicate number of participants with missing data for each variable of interest | N/A |
| Outcome data | 15* | Report numbers of outcome events or summary measured | Results, paragraph 2-4 |
| Main results | 16 | (*a*) Give unadjusted estimates and, if applicable, confounder-adjusted estimates and their precision (eg, 95% confidence interval). Make clear which confounders were adjusted for and why they were included | Results, paragraph 2-4 |
|  |  | (*b*) Report category boundaries when continuous variables were categorized | N/A |
|  |  | (*c*) If relevant, consider translating estimates of relative risk into absolute risk for a meaningful time period | N/A |
| Other analyses | 17 | Report other analyses done—eg analyses of subgroups and interactions, and sensitivity analyses | Discussion, paragraph 5 |
| Discussion | | |  |
| Key results | 18 | Summarise key results with reference to study objectives | Discussion, paragraph 1 |
| Limitations | 19 | Discuss limitations of the study, taking into account sources of potential bias or imprecision. Discuss both direction and magnitude of any potential bias | Discussion, paragraph 5 |
| Interpretation | 20 | Give a cautious overall interpretation of results considering objectives, limitations, multiplicity of analyses, results from similar studies, and other relevant evidence | Discussion, paragraph 2-4 |
| Generalisability | 21 | Discuss the generalisability (external validity) of the study results | Discussion, paragraph 5 |
| Other information | | |  |
| Funding | 22 | Give the source of funding and the role of the funders for the present study and, if applicable, for the original study on which the present article is based | Methods, paragraph 9 |

*Give information separately for exposed and unexposed groups.

**Note:** An Explanation and Elaboration article discusses each checklist item and gives methodological background and published examples of transparent reporting. The STROBE checklist is best used in conjunction with this article (freely available on the Web sites of PLoS Medicine at http://www.plosmedicine.org/, Annals of Internal Medicine at http://www.annals.org/, and Epidemiology at http://www.epidem.com/). Information on the STROBE Initiative is available at www.strobe-statement.org.
